# Cationic Carrier Mediated Delivery of Anionic Contrast Agents in Low Doses Enable Enhanced Computed Tomography Imaging of Cartilage for Early Osteoarthritis Diagnosis

**DOI:** 10.1021/acsnano.2c12376

**Published:** 2023-03-29

**Authors:** Chenzhen Zhang, Armin Vedadghavami, Tengfei He, Julia F. Charles, Ambika G. Bajpayee

**Affiliations:** † Department of Bioengineering, Northeastern University, 360 Huntington Avenue, Boston, Massachusetts 02115, United States; ‡ Department of Orthopaedic Surgery, Brigham and Women’s Hospital, 60 Fenwood Road, Boston, Massachusetts 02115, United States

**Keywords:** cationic carriers, contrast enhanced computed tomography, cartilage, imaging, osteoarthritis, diagnosis, electrostatic interactions

## Abstract

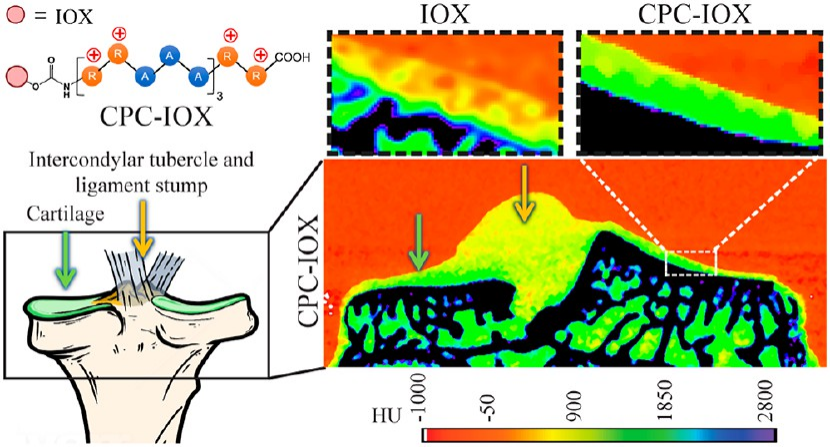

Cartilage tissue exhibits early degenerative changes with onset of osteoarthritis (OA). Early diagnosis is critical as there is only a narrow time window during which therapeutic intervention can reverse disease progression. Computed tomography (CT) has been considered for cartilage imaging as a tool for early OA diagnosis by introducing radio-opaque contrast agents like ioxaglate (IOX) into the joint. IOX, however, is anionic and thus repelled by negatively charged cartilage glycosaminoglycans (GAGs) that hinders its intra-tissue penetration and partitioning, resulting in poor CT attenuation. This is further complicated by its short intra-tissue residence time owing to rapid clearance from joints, which necessitates high doses causing toxicity concerns. Here we engineer optimally charged cationic contrast agents based on cartilage negative fixed charge density by conjugating cartilage targeting a cationic peptide carrier (CPC) and multi-arm avidin nanoconstruct (mAv) to IOX, such that they can penetrate through the full thickness of cartilage within 6 h using electrostatic interactions and elicit similar CT signal with about 40× lower dose compared to anionic IOX. Their partitioning and distribution correlate strongly with spatial GAG distribution within healthy and early- to late-stage arthritic bovine cartilage tissues at 50–100× lower doses than other cationic contrast agents used in the current literature. The use of contrast agents at low concentrations also allowed for delineation of cartilage from subchondral bone as well as other soft tissues in rat tibial joints. These contrast agents are safe to use at current doses, making CT a viable imaging modality for early detection of OA and staging of its severity.

Osteoarthritis (OA) is a complex debilitating disease affecting millions of people worldwide, causing loss of joint function, productivity, and quality of life. OA attacks the entire joint including cartilage, meniscus, ligaments, synovium, and the bone.^1,2^ Current preclinical research has focused on development of disease modifying OA drugs (DMOADs) that have shown effectiveness in reversing or inhibiting OA but only if administered at *early stages of the disease*, before the joints become symptomatic.^3,4^ Early-stage detection of OA, however, remains elusive. By the time OA associated loss of tissue is radiographically evident (by measuring joint space narrowing using X-ray), major changes in the biochemical composition and morphology of soft tissues like articular cartilage have already occurred.^5^

Non-mineralized soft tissues can now be imaged with 3D X-ray based contrast enhanced computed tomography (CECT) by using radio-opaque, anionic contrast agents that help compensate for their poor radiopacity.^6,7^ CT imaging is desirable due to its lower cost and high spatial resolution over short acquisition times, and it can be used for simultaneous imaging of other tissues like meniscus and subchondral bone along with cartilage.^8^ Cartilage extracellular matrix, however, is negatively charged comprising a complex meshwork of Collagen II filled with densely packed negatively charged aggrecan–glycosaminoglycans (GAGs), whose density increases with depth into the tissue. This hinders transport and penetration of most macromolecules administered in synovial fluid (Figure 1A). Currently available contrast agents are small anionic molecules and thus are non-targeted and repelled by the negatively charged cartilage, which hinders their intra-tissue penetration and partitioning resulting in poor CT attenuation.^6,7,9^ This is further complicated by their short intra-tissue residence time owing to rapid clearance *via* lymphatics and vasculature following their direct intra-articular injection into the joint. High doses of contrast agents are therefore required to enhance initial flux and achieve intra-cartilage concentrations sufficient for CT imaging that can, however, cause local and systemic toxicity.^10,11^ Levenston and colleagues used Hexabrix, a clinically available CT contrast agent containing ioxaglate (IOX), a negatively charged hexaiodinated dimer, for CT imaging of cartilage. This yielded an equilibrium distribution of ionic contrast agent which is inversely related to the density of negatively charged GAGs.^12^ A high 128 mg of (iodine) I/mL of IOX was used that elicited sufficient X-ray attenuation for cartilage imaging but also resulted in overlapping signals from the highly mineralized bone. Additionally, its application could not be translated *in vivo* owing to rapid clearance of IOX from the joint preventing adequate intra-cartilage equilibration. Thereafter, IOX was also used for peripheral quantitative CT detection of spatial aggrecan content in cartilage. 16 mg of I/mL of IOX produced a high CT signal in trypsin induced aggrecan depleted cartilage explants, but IOX diffused evenly throughout the tissue, impairing detection of any aggrecan concentration gradients.^13^ Initial stages of OA are associated with loss of GAGs from cartilage, which increasingly gets worse as the disease progresses, affecting the tissue’s structure, electromechanical properties, and its function.^14−16^ A contrast agent that can enable CT imaging in low doses to detect changes in aggrecan content and its spatial distribution can help early diagnosis of OA, enable staging of disease severity, and ultimately provide opportunities for timely therapeutic intervention. This can be helpful especially in monitoring patients with traumatic joint injuries who are at a high risk of developing OA in future.^17^ Our laboratory has designed cartilage targeting optimally charged cationic carriers that possess sufficient electrical driving force to rapidly carry the conjugated cargo through the full thickness of cartilage tissue in high concentrations following their intra-articular administration in the synovial joint.^18−22^ Here we use an arginine rich cationic peptide carrier with net charge of +8 (CPC, (RRAAAA) _3_RR)^19^ and a cationic multi-arm PEGylated Avidin (mAv) nanoconstruct^20,23,24^ for intra-cartilage delivery of IOX as both carriers can rapidly penetrate through the full thickness of healthy and OA cartilage resulting in about 100× higher uptake than uncharged carriers. These carriers are designed to possess optimal cationic charge based on cartilage tissue’s negative fixed charge density (FCD) that enables high Donnan partitioning (*K*_CPC_ = 7.6; *K*_mAv_ = 6) at the synovial fluid–cartilage interface resulting in steep intra-tissue concentration gradients and thus facilitating rapid transport and high uptake.^25−27^ Too high of a cationic charge on the carrier can result in strong binding with cartilage GAGs in the superficial zone, preventing their penetration (Figure 1B). The carriers used here take advantage of weak-reversible electrostatic binding with negatively charged GAGs to penetrate through the full thickness of tissue.

**Figure 1:**
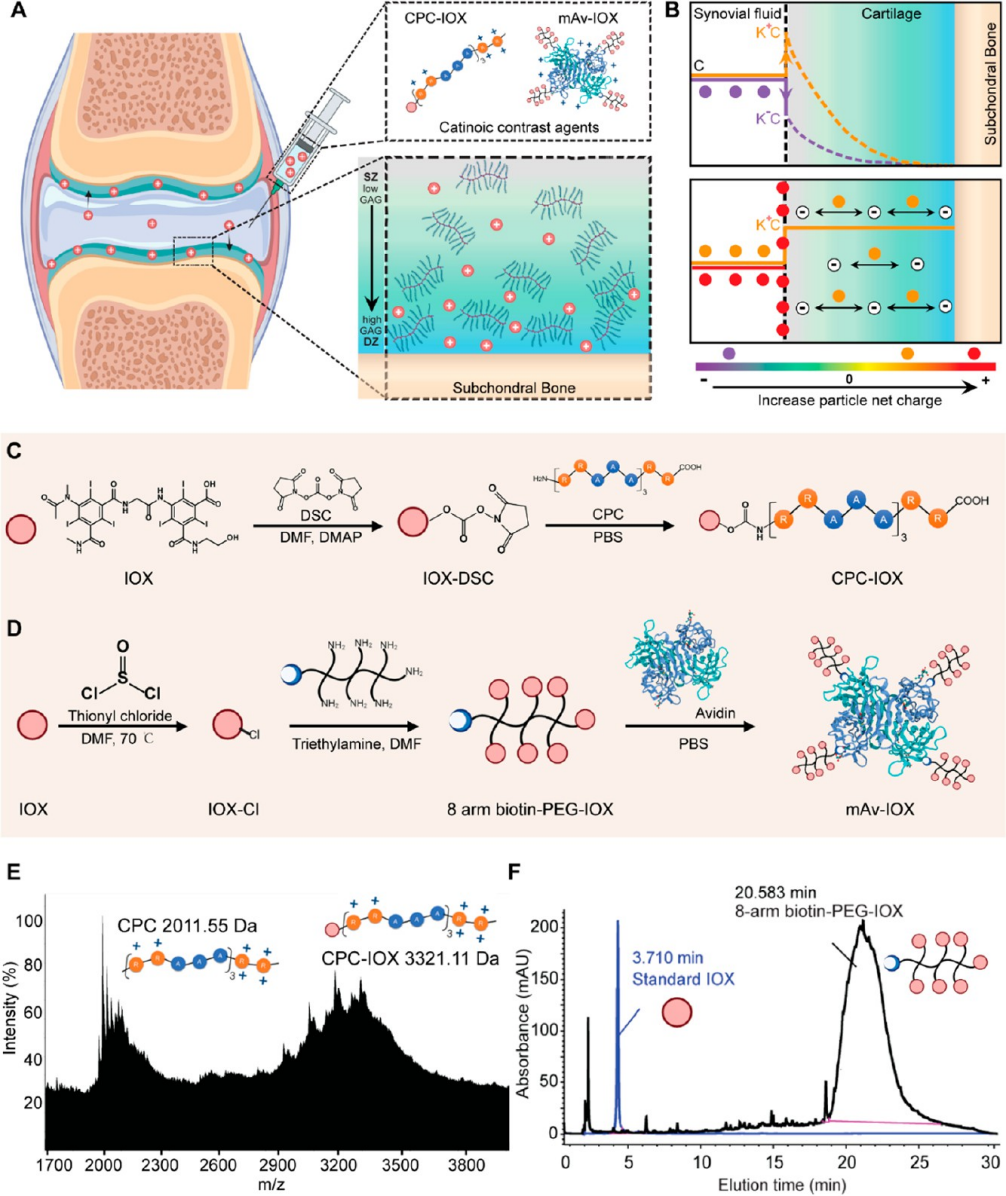
(A) Intra-articular injected ioxaglate (IOX) based cationic contrast agents, CPC–IOX and mAv–IOX, rapidly penetrate through the full thickness of cartilage in high concentrations by using weak-reversible nature of charge interactions with cartilage residing aggrecan-glycosaminoglycans (GAGs). (B) CPC and mAv chosen as cationic carriers due to their high Donnan partitioning (K^+^) that enhances their concentration from C to K^+^C at the synovial fluid–cartilage interface, resulting in steep concentration gradients and thus faster intra-tissue diffusion rates. Conversely, negatively charged molecules (like IOX) may partition down from C to K^–^C, explaining lower transport rates and intra-cartilage uptakes. Optimal net charge on carriers is critical to enable sufficient electrical driving force to carry the conjugated contrast agent with them into the cartilage; too high of a positive charge can make carriers stick to the cartilage surface. Reaction schemes for synthesis of cationic contrast agents, (C) CPC–IOX and (D) mAv–IOX. (E) MALDI-TOF confirmation of the increased molecular mass of conjugated CPC–IOX from 2011.55 Da for CPC to 3321.11 Da for CPC–IOX, implying 1:1 CPC to IOX conjugation by mole. (F) HPLC confirmed the shift peak of 8-arm biotin–PEG–IOX in mAv–IOX from 3.710 min (standard IOX peak) to 20.583 min following conjugation. An average of 3.82 ± 0.50 IOX per mole of 8-arm biotin–PEG was estimated.

We report the development of cationic contrast agents by conjugating CPC and mAv to IOX whose partitioning and distribution within cartilage correlates strongly with its spatial GAG content. We demonstrate their effectiveness in enabling high resolution CECT imaging of cartilage to detect changes in GAG distribution within healthy and early- to late-stage arthritic bovine cartilage tissues at 50–100× lower doses of IOX compared to that used in the current literature.^28−30^ Their use at low concentration enables delineation of cartilage from subchondral bones as well as other soft tissues in rat tibial joints. The contrast agents are safe to use, making CECT a viable imaging modality for early OA detection and monitoring. It can find applications especially in monitoring high risk patients with a history of traumatic injuries, for defining patient phenotype and deciding treatment options as well as for evaluating the effectiveness of pharmacological therapies in clinical trials.

## Results

### CPC-IOX Synthesis

The intermediate product, IOX–DSC, was confirmed using MALDI as the mass increased from 1065.8 Da for IOX to 1291.7 Da following successful reaction of DSC with the hydroxyl group of IOX (Supporting Information Figure S1A). This was also confirmed by HPLC as the elution peak shifted from 10.39 min for IOX to 15.77 min for IOX–DSC (Figure S1B). Finally, MALDI-TOF also showed an increase in mass from 2011 Da for CPC to 3321 Da following conjugation of IOX–DSC to the N-terminus of CPC (Figure 1E), confirming conjugation of CPC to IOX in 1:1 molar ratio. Following conjugation, the net charge is expected to reduce from +8 for CPC to +7 for CPC–IOX due to negative one charge of ioxaglate.^31^

### mAv-IOX Synthesis

The carboxylic acid on the benzene ring does not undergo simple nucleophilic substitutions due to the electron withdrawing property of three iodines in the aryl halide structure of IOX.^32^ This makes the benzoic acid group of IOX less reactive and thus less conducive for the common EDC/NHS or DCC/NHS coupling reactions for conjugating IOX to 8-arm PEG *via* the formation of an amide bond. Instead, thionyl chloride can react with carboxyl group in IOX, forming a highly reactive acid chloride (IOX-Cl) intermediate with low solubility (Figure 1D).^33^ Its solubility is improved following conjugation to 8-arm PEGs. Compared to the ^1^H NMR spectra of IOX (Figure S2A), activation of benzoic acid in IOX–Cl resulted in the disappearance of peak *a* at 10.5 ppm (−COOH) and peak *d* at 4.26 ppm (−OH). Also, the shift in peaks of the methylene group (peaks *b* and *c*) adjacent to the hydroxyl group confirmed that the IOX chemical structure had changed following reaction with thionyl chloride.

Biotinylation of 8-arm PEG^20^ showed an increase in mass by 282 Da, confirming an average 1.15 biotin per PEG (Figure S2C). HPLC analysis revealed a delayed elution time of 8-arm biotin-PEG–IOX at 20.583 min, while the IOX standard eluted earlier at 3.710 min (Figure 1F). Since the low UV absorption of PEG polymer in HPLC would not interfere with UV readings of IOX, the area under the curve for 8-arm biotin–PEG–IOX was used to estimate IOX loading at an average of 3.82 ± 0.50 IOX mol/PEG. MALDI-TOF MS also confirmed the increase in mass of 8-arm biotin–PEG–IOX from 10902 to 13820 Da, estimating an average of 2.33 IOX mol/PEG (Figure S2B,C). However, since IOX conjugation to PEG increases its polydispersity index, MALDI-TOF MS is not ideal as its accuracy is known to be best for polymers with low polydispersity.^34^ Thus, IOX loading estimated using HPLC was used for the next experiments.

IOX loaded PEGs were conjugated to Avidin to form multi-arm Avidin–IOX (mAv–IOX), which was confirmed using HABA dye assay and UPLC (Figure S2D) as described previously.^20,35^ The final product mAv–IOX, therefore, had about 15 IOX mol per Avidin. The ζ potential of mAv–IOX reduced to 8.69 ± 0.7 mV due to loading of anionic IOX (ζ potential of unmodified Avidin was measured as 18.3 ± 0.5 mV). The average hydrodynamic diameter of mAv was estimated as ∼8.1 nm.^20^ After conjugation with IOX, its diameter increased to 9.64 ± 2.13 nm with a low polydispersity index (PDI) of 0.083 ± 0.027 measured using a particle analyzer (Litesizer 500, Anton Paar, Austria).

### Cationic Carriers Penetrated through the Full Thickness of Rat Knee Joint Tissues

**Figure 2:**
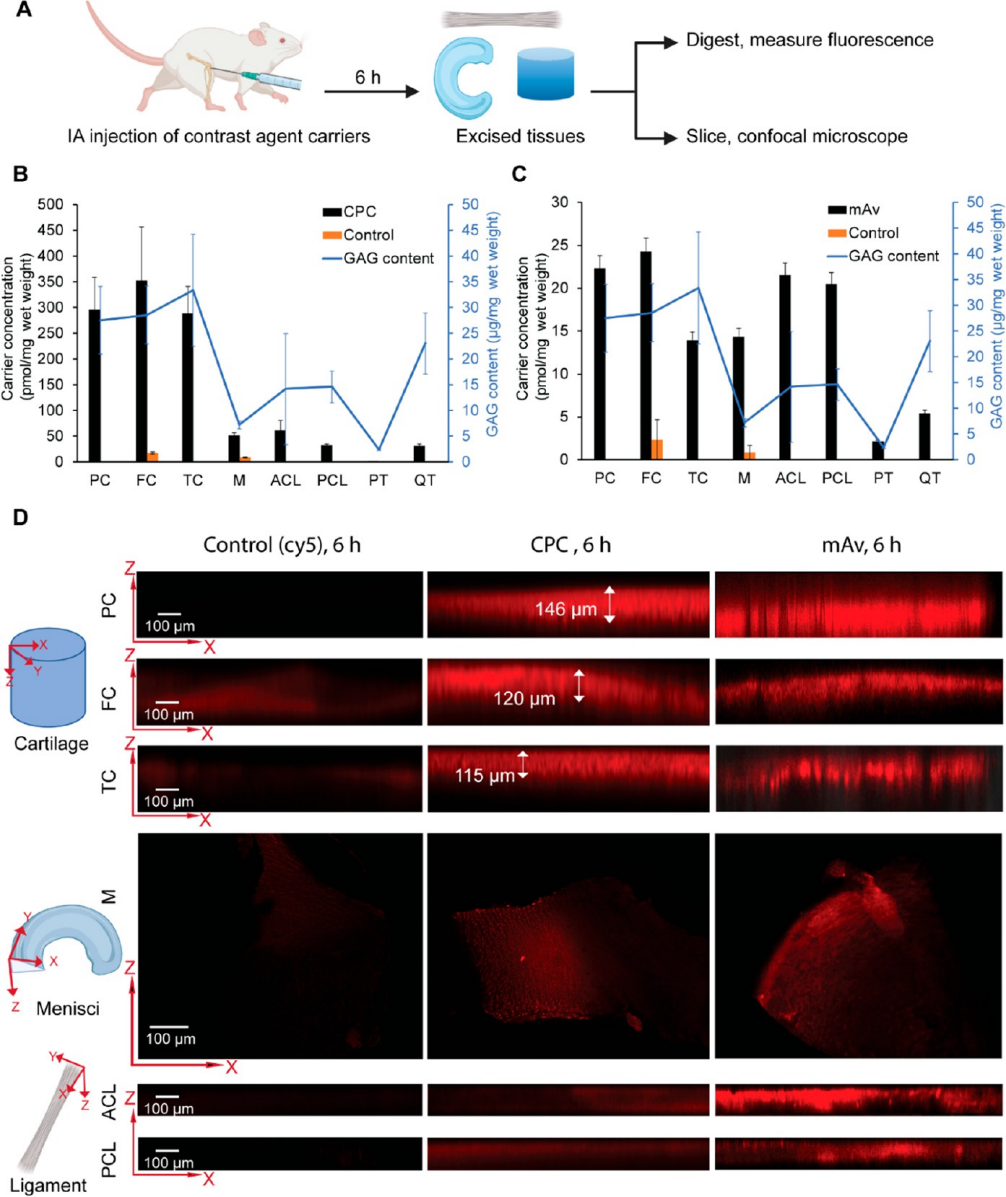
(A) *In vivo* biodistribution of cy5-labeled CPC, Texas-Red-labeled mAv, and free dye (cy5 or Texas-Red) after their intra-articular administration in healthy rat knee joints at 6 h. (B) CPC and (C) mAv resulted in the highest uptake in articular cartilage (PC, FC, and TC). mAv was also uptaken in menisci and ligaments (M, ACL, and PCL). GAG content in respective tissues is shown in blue. (D) Confocal microscopy images shown in *X*–*Z* plane confirm the fluorescence intensity and distribution of the dye alone, CPC and mAv through the full thickness of cartilage, menisci, and ligament tissues.

Before conjugating to IOX, *in vivo* distribution of cationic carriers (CPC and mAv) in healthy rat knee joints was investigated to evaluate their tissue targeting properties and depth of penetration within a short period of 6 h following their intra-articular (IA) administration (Figure 2A). Both CPC and mAv were uptaken in highest concentrations by the articular cartilage (patellar (PC), femoral (FC), and tibial (TC)) followed by that in menisci tissues (M); the least amount was present in tendons, which is consistent with tissues’ respective GAG content, confirming the dominant role of electrostatic interactions between cationic carriers and the negatively charged aggrecan GAGs (Figure 2B,C; GAG content in respective tissues is shown in blue). Anterior and posterior cruciate ligaments (ACL and PCL) unexpectedly showed high mAv uptake despite their lower GAG content, which may be due to their close proximity to the site of injection or can be attributed to higher tissue matrix porosity allowing for enhanced solute diffusivity^36,37^ or mAv’s high hydrophilicity due to the presence of branched PEGs that can further enhance its affinity to intra-tissue hydrophilic macromolecules like aggrecan–GAGs and collagen meshwork.^27^ PEGylation is also known to stabilize the protein structure, making it less susceptible to enzymatic degradation and further enhancing its longevity in the complex joint environment.^27^ This is corroborated by our finding that, at 6 h, we measured at least 33 ± 1.6% injected mAv to be present inside the joint space, while only about 7.9 ± 0.4% injected CPC was found to be present. Confocal microscopy images (Figure 2D) confirmed that both carriers penetrated through the full thickness of articular cartilage, menisci, and ligament tissues within 6 h. It is important to note that free dyes (both Cy5 and Texas-Red) cleared out rapidly from the joint space and negligible fluorescence was observed at 6 h in all joint tissues.

### CT Attenuation from Cationic Contrast Agents Achieves Intra-cartilage Equilibration within 6 h

**Figure 3:**
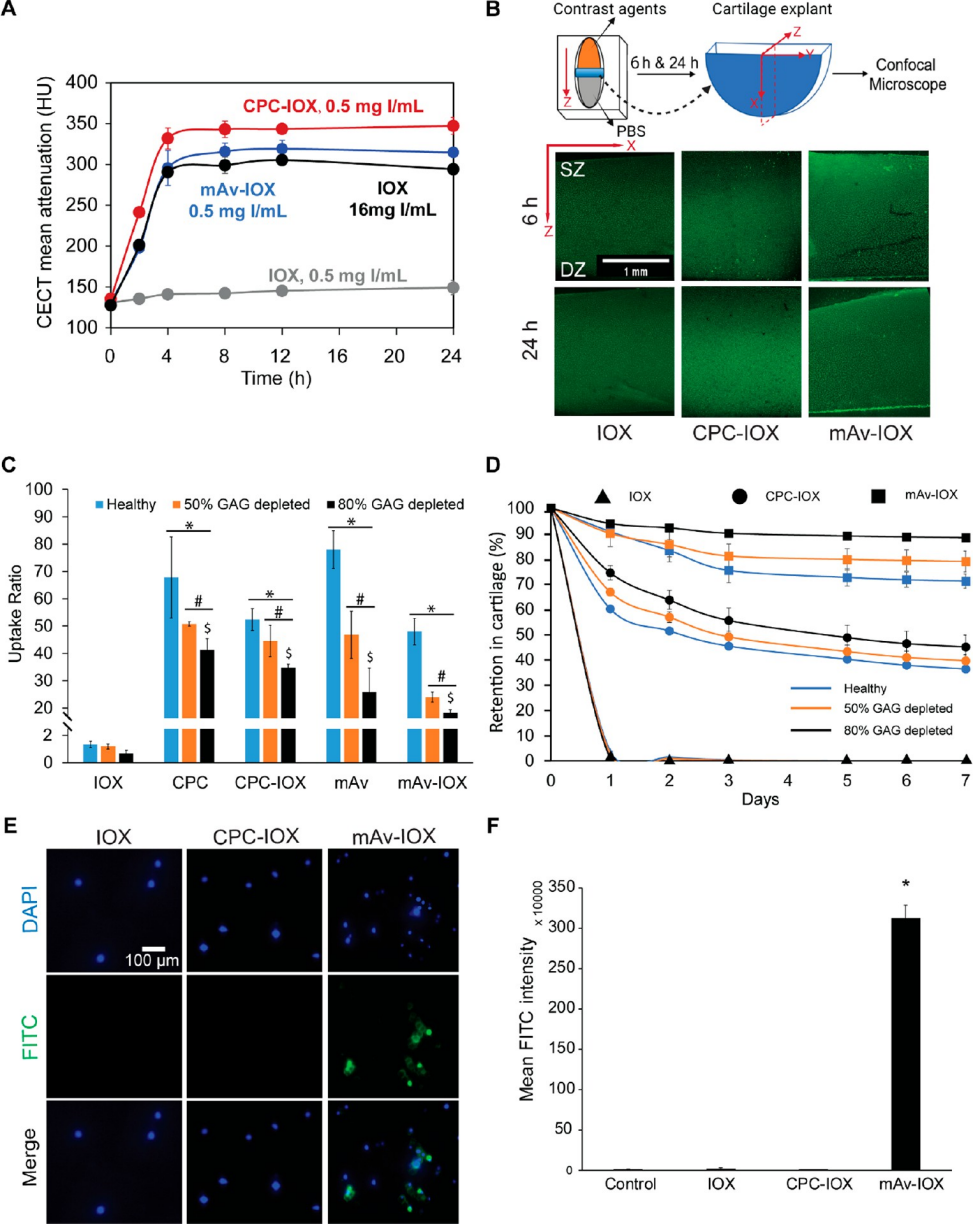
(A) IOX, CPC–IOX, and mAv–IOX achieve intra-cartilage equilibration within 4–8 h. (B) Confocal microscopy images show full-thickness penetration of IOX, CPC–IOX, and mAv–IOX within 6 h. (C) Intra-cartilage equilibrium uptake of IOX, CPC, CPC–IOX, mAv, and mAv–IOX in healthy, 50% GAG depleted, and 80% GAG depleted bovine cartilage explants measured at 6 h (* *vs* respective condition in IOX group; # *vs* respective condition in healthy; $ *vs* 50% GAG depleted; *p* ≤ 0.05). (D) % retention of IOX, CPC–IOX, and mAv–IOX in healthy, 50% GAG depleted, and 80% GAG depleted cartilage samples when desorbed in PBS for 7 days. Chondrocyte uptake of FITC-labeled IOX, CPC–IOX, and mAv–IOX analyzed using (E) fluorescence imaging (blue, cell nuclei; green, contrast agent) and (F) flow cytometry (* *vs* untreated control; *p* ≤ 0.05).

CECT mean attenuation of cationic contrast agents and the time course over which equilibration was achieved was determined and compared with that of anionic IOX. CECT attenuation from CPC–IOX and mAv–IOX reached equilibration between 4 and 8 h, which was similar to that of anionic IOX (Figure 3A). However, CPC–IOX and mAv–IOX at a low concentration of 0.5 mg of I/mL showed significantly higher CECT mean attenuation than an equivalent concentration of IOX. 32 times higher concentration of IOX (16 mg of I/mL) was required to match the equilibrium attenuation of cationic contrast agents. It is important for contrast agents to diffuse rapidly through cartilage tissue in high concentrations following their intra-articular administration in patient knee joints for CT imaging to be clinically feasible. Confocal imaging revealed that both CPC–IOX and mAv–IOX penetrated through the full thickness of cartilage explants within 6 h, similar to unmodified IOX (Figure 3B). No difference in fluorescence intensity of labeled contrast agents was observed at 6 and 24 h confirming that equilibration was achieved within 6 h.

### Cationic Contrast Agents Show 20–50× Higher Equilibrium Uptake in Healthy and Arthritic Cartilage Compared to Anionic IOX

Cationic contrast agents, CPC–IOX and mAv–IOX, showed about 50× higher uptake in healthy cartilage. Their uptake reduced significantly in arthritic cartilage compared to healthy yet remained 20–45× higher compared to IOX alone, implying that these cationic contrast agents can be used for diagnosis of early OA and staging of its severity (Figure 3C).Similarly, intra-cartilage equilibrium uptake of cationic carriers, CPC and mAv, was 30–80× higher than that of IOX in healthy as well as 50% and 80% GAG depleted cartilage explants.

Retention studies showed that 50–70% uptaken CPC–IOX is retained within healthy and GAG-depleted cartilage explants over 48 h desorption in PBS while the majority of mAv–IOX is retained within cartilage over 7 day desorption owing to electrostatic binding interactions (Figure 3D), which is consistent with desorption results of cationic carriers alone (Figure S3). All of the uptaken IOX, however, desorbed within 24 h of desorption. Our previous work shows that mAv is uptaken by chondrocytes,^27^ consistent with our current findings where mAv–IOX was also uptaken by the chondrocytes (Figure 3E-F). Since CPC–IOX is not uptaken by chondrocytes and can desorb out of cartilage (implying faster elimination from the joint following CT imaging), it offers itself as a more promising contrast agent than mAv–IOX for cartilage CT imaging.

### Low Doses of Cationic Contrast Agents Enable CECT Imaging of Healthy and Arthritic Bovine Cartilage Explants Allowing for High Resolution Spatial GAG Mapping

**Figure 4:**
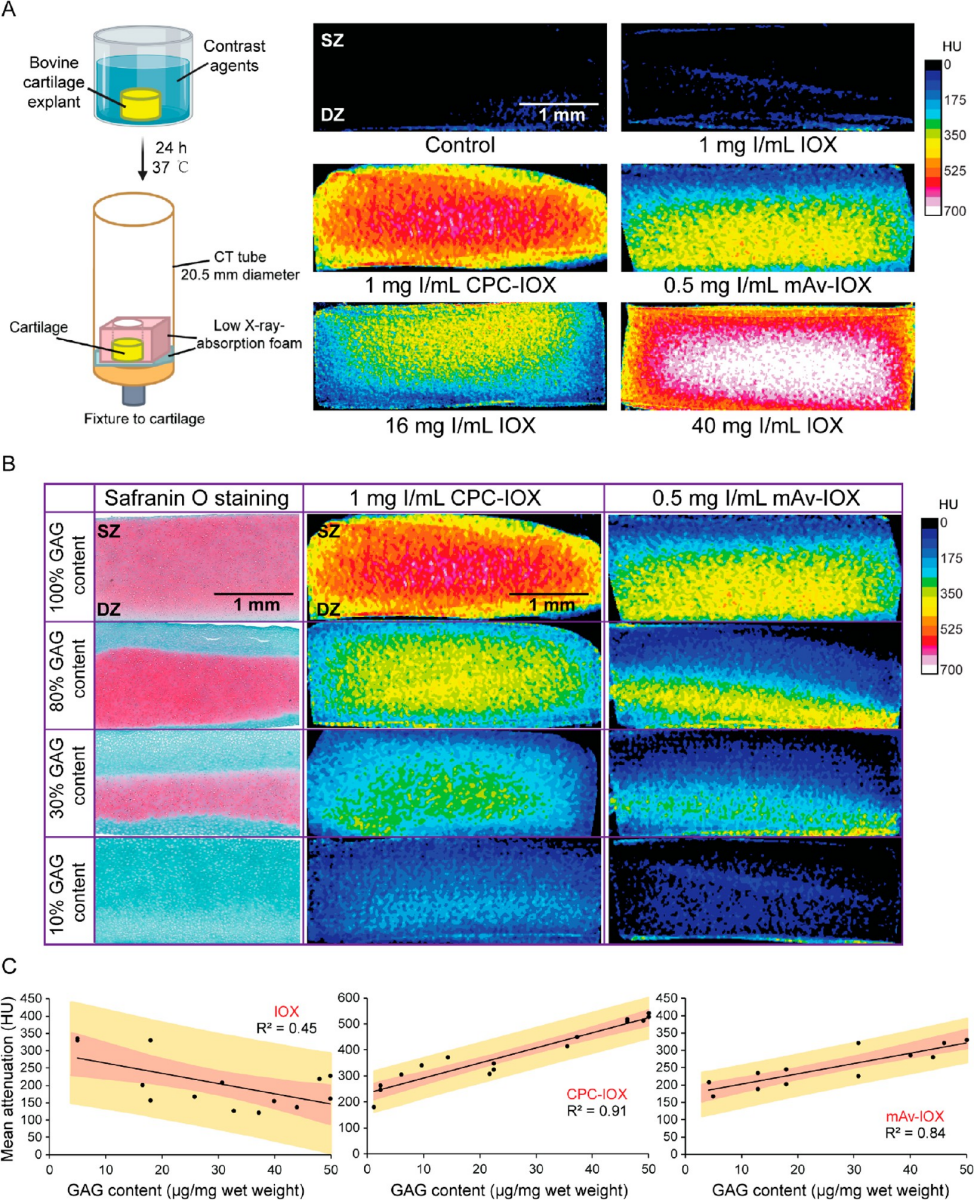
(A) 2D color map of cartilage μCT images using IOX, CPC–IOX, and mAv–IOX contrast agents. CPC–IOX and mAv–IOX show significantly higher CECT attenuation than IOX at equivalent concentrations. A high 16–40 mg of I/mL of IOX concentration is required to match the effects of enhanced CT attenuation of CPC–IOX or mAv–IOX. (B) Safranin-O staining showing spatial GAG distribution in bovine cartilage explants with 10, 30, 80, and 100% GAG content. Corresponding CT color maps following treatment with CPC–IOX and mAv–IOX are shown demonstrating their ability to predict spatial distribution of GAGs in 1 mm thick bovine healthy and arthritic cartilage explants. (C) Mean CECT attenuation achieved using CPC–IOX (*R*^2^ = 0.91) and mAv–IOX (*R*^2^ = 0.84) correlates positively and strongly with cartilage GAG content. However, mean attenuation with IOX showed weak and low correlation with tissue GAG content (*R*^2^ = 0.45). The red shaded curved region shows the 95% confidence intervals for individual points, and the yellow shaded curve region shows the 95% prediction intervals.

A low 0.5–1 mg of I/mL of CPC–IOX and mAv–IOX resulted in high CT attenuation (Figure 4A), while IOX at an equivalent concentration of 1 mg of I/mL did not show any CT signal, similar to blank control. A 32–40× higher concentration of anionic IOX was needed to match the CT signal obtained with cationic contrast agents (see color map images of cartilage treated with 16 and 40 mg of I/mL of IOX). Both cationic contrast agents resulted in enhanced CT signal in full-thickness cartilage explants with 30–100% GAG content that correlated with the spatial GAG distribution in cartilage explants as shown by Safranin O staining (Figure 4B), further confirming the potential of these cationic contrast agents in diagnosing different stages of OA. The mean CT attenuation from the entire segmented volume of healthy and varying degrees of GAG depleted cartilage explants were plotted against their respective GAG content to estimate correlations (Figure 4C). A weak and negative correlation (*R*^2^ = 0.45) was observed for CT attenuation from IOX and cartilage GAG content owing to its negative charge, which is repelled by the negatively charged cartilage GAGs. Cationic CPC–IOX and mAv–IOX showed strong and positive correlations (*R*^2^ = 0.91 and 0.84 respectively) between mean CT attenuation and cartilage GAG content, further confirming their use for diagnosing early and different stages of OA.

### CPC–IOX is Most Effective in Identifying and Delineating Cartilage from Other Soft Tissues and the Subchondral Bone in Rat Tibial Joints

**Figure 5:**
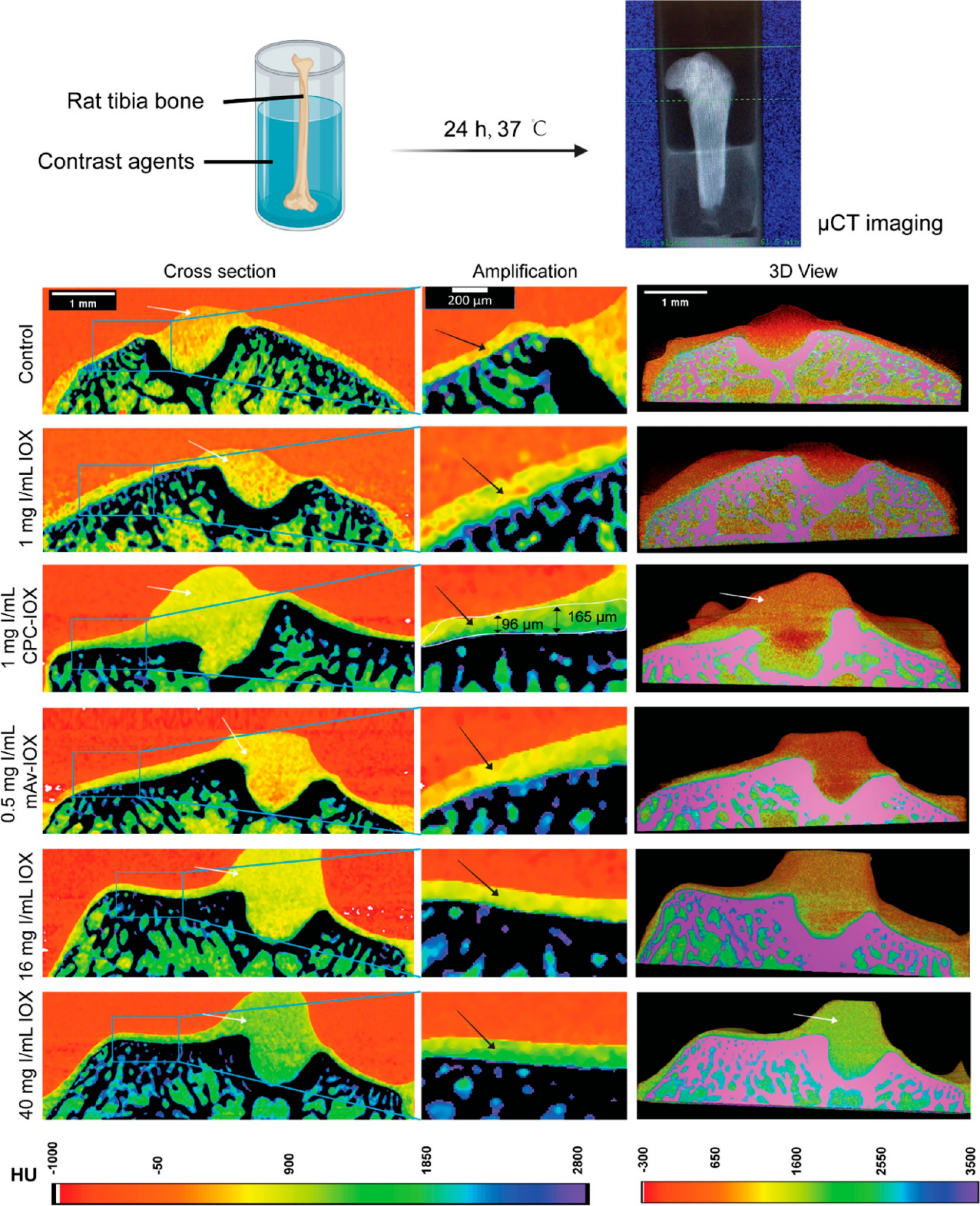
*Ex vivo* CECT images of excised rat tibial bones using IOX, CPC–IOX, and mAv–IOX contrast agents. The left column shows the cross-section color map of CT images of excised rat tibia. The middle column shows the magnified region of tibial bone with cartilage tissue for clarity. The right column shows the 3D view of rat tibial bones. White arrows indicate soft tissue (partial ligaments or muscle remaining on the surface of tibial groove). Black arrows indicate articular cartilage.

As shown in color maps (Figure 5), 1 mg of I/mL of CPC–IOX generated CT attenuation with high signal-to-noise ratio, enabling differentiation of cartilage (black arrows) from the subchondral bone and other soft tissues like the ligaments (white arrows); the thickness of delineated cartilage (green in the color map) was estimated to be 96–165 μm. An equivalent concentration of 1 mg of I/mL of IOX resulted in similar results as the saline control, where cartilage tissue was not identifiable. A high 40 mg of I/mL dose of IOX, however, enabled high CT attenuation (green color) from both cartilage and other soft tissues due to non-specific absorption of IOX by all soft tissues. A low concentration of 0.5 mg of I/mL of mAv–IOX did not produce a sufficient CT signal for effective cartilage imaging. Its use at high concentration was limited by solubility issues. Similarly, 16 mg of I/mL of IOX produced insufficient CT signal (yellow) to clearly delineate cartilage from other soft tissues. 3D reconstructions are also presented; delineation of cartilage tissue (green color) from the surface of the tibial bone as well as from other soft tissues is distinctly seen in the 1 mg of I/mL of CPC–IOX and the high 40 mg of I/mL of IOX conditions (in this case, however, cartilage is indistinguishable from the ligament stump). Therefore, CPC–IOX at a low dose of 1 mg of I/mL was most effective in identifying and delineating cartilage from other soft tissues and the subchondral bone of rat tibial joints.

### Chondrocyte Cytotoxicity and Cartilage Tissue Health

**Figure 6:**
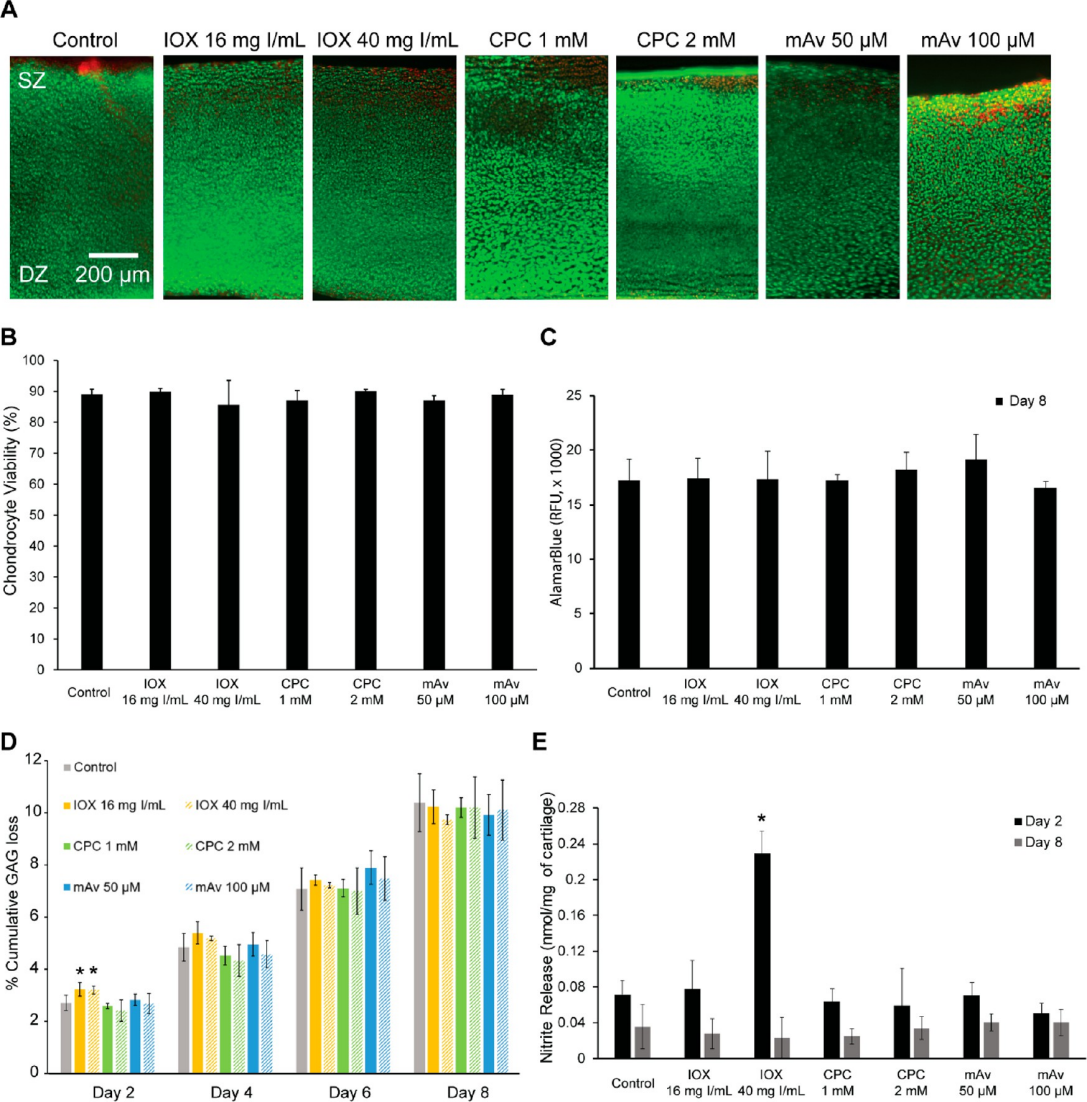
Chondrocyte viability after 8 days of culture. (A) Live/dead staining revealing that there was no cytotoxicity observed when treated with IOX, CPC, or mAv at working or 2× higher concentration. Green fluorescence indicates viable cells, and red fluorescence indicates dead cells. (B) Quantitative evaluation of chondrocyte viability in live/dead staining using ImageJ. (C) Chondrocyte metabolism measured as relative fluorescence units (RFU) using AlamarBlue assay on day 8. (D) % cumulative GAG loss released from cartilage to culture media measured over 8 days following treatment with contrast agents (* *vs* untreated control; *p* ≤ 0.05). (E) Nitrite released to media per milligram of cartilage tissue on day 2 and day 8 (* *vs* untreated control; *p* ≤ 0.05).

Working concentrations of 1 and 0.5 mg of I/mL for CPC–IOX and mAv–IOX require 1 mM CPC and 50 μM mAv for μCT imaging experiments, respectively. Therefore, cell toxicity and cartilage tissue health were evaluated following treatment with up to 2× higher working concentrations (Figure 6). No differences in chondrocyte live–dead staining and metabolism rates were seen across different concentrations of CPC and mAv compared to the untreated control condition (Figure 6A–C). Note that some cell death in the superficial zone is typical depending on the location of harvesting along the joint. Also, excision of tissues using punches can contribute to cell death.^20,38−40^ Over 8 days of culture, no significant difference in levels of GAG released from CPC-treated, mAv-treated, or untreated groups was observed (Figure 6D). However, treatment with high dose IOX (both at 16 and 40 mg of I/mL) resulted in significantly higher GAG release on day 2. Total GAG content in cartilage explants at the end of the culture was measured by adding the total amount released to the media and that remaining within the explant and was found to be similar across all treatment conditions, indicating no change in GAG synthesis levels (Figure S5). CPC and mAv treatments did not elicit any inflammatory response as shown by nitrite release on day 2 and day 8 (Figure 6E). IOX at 40 mg I/mL, however, resulted in 3× higher level of nitrites at day 2 of culture.

## Discussion

Contrast agents that can enable detection of intra-cartilage aggrecan content, its spatial distribution, and changes in tissue morphology in low doses can make CECT a promising imaging option for early OA detection and monitoring. Here we engineer optimally charged cationic contrast agents based on cartilage negative FCD, such that they can equilibrate and penetrate through the full thickness of cartilage within 6 h using electrostatic interactions resulting in 20–50× higher uptake than unmodified anionic IOX. As a result, high CECT attenuation can be achieved with very low IOX concentration (0.5–1 mg of I/mL) which is 50–100× lower compared to that used in the current literature.^28−30^ This is important as it can significantly reduce toxicity concerns^41−43^ associated with high dose IOX and enable its clinical use. Additionally, CECT attenuation from equilibrated cationic contrast agents within cartilage correlated strongly with the spatial GAG distribution within the full thickness of healthy and early- to late-stage arthritic cartilage, as shown by Safranin-O staining (Figure 4B). Furthermore, a strong positive correlation was observed between mean CT attenuation from cationic contrast agents and total GAG content in chondroitinase induced to a varying degree of GAG depleted cartilage explants (*R*^2^ for CPC–IOX = 0.91; *R*^2^ for mAv–IOX = 0.84). This illustrates their potential applications in early detection of OA and for quantitative staging of its severity. Equivalent concentration of IOX did not elicit any CT signal.

The two cationic carriers, CPC and mAv, are designed to achieve a step jump in their concentration at the cartilage–synovial fluid interface by a high Donnan partitioning factor (*K*) between 6 and 7 and take advantage of weak-reversible electrostatic interactions with cartilage aggrecan–GAGs (Figure 1A).^18,19^ This results in steep intra-cartilage concentration gradients explaining their rapid transport through the full thickness of rat joint tissues within 6 h following their intra-articular administration. Anionic contrast agents, on the other hand, suffer from low intra-cartilage uptake that would require cycling or other physical activity post-administration to add convective driving forces to enhance uptake. However, dynamic loading induced convective flow is known to increase transport by only about 2-fold for large macromolecules,^44,45^ while the electrostatic effects described here enable increase in intra-cartilage concentration by 20–50× eliciting similar CT signal with 32–40× lower concentration of cationic contrast agents than unmodified anionic IOX. Articular cartilage, which has the highest GAG concentration, exhibited the greatest carrier uptake compared to other rat joint soft tissues. This selective uptake depending on a tissue’s negative FCD and ability to image at low concentrations of CPC–IOX enabled effective delineation of cartilage tissue from the high mineralized subchondral bone and other soft tissues (Figure 5). IOX, at similar concentration, functioned the same as saline control. At a high 40 mg of I/mL of concentration, IOX enabled cartilage imaging but since it was also nonselectively absorbed by other tissues, cartilage remained indistinguishable from other soft tissues like the ligament stump present on rat tibial bone.

Grinstaff and colleagues developed cationic iodinated contrast agent, CA4^+^ with +4 net charge that exhibited 8-fold greater uptake than anionic IOX in bovine osteochondral plugs.^9,11,33,46^ As a result of this, 12 mg of I/mL of CA4^+^ showed similar CECT attenuation as from 80 mg of I/mL of anionic IOX and demonstrated strong positive correlation with GAG content.^31,33,47^ In comparison, optimally charged CPC–IOX resulted in 20–50× higher intra-cartilage uptake and at low 1 mg of I/mL dose exhibited CECT attenuation similar to that of 40 mg of I/mL of IOX with strong positive correlation with GAG content. Recently, a cationic tantalum oxide nanoparticle (NP) was designed that showed high CECT correlation with cartilage GAG content but required long equilibrium time (24 h) and high NP concentration of 80 mg/mL.^48^ Use of mAv–IOX was limited by Avidin’s solubility as it started to aggregate at concentrations higher than 0.5 mg of I/mL. The branched PEG structure of mAv, however, offers 28 sites for IOX loading.^20,35^ In the current design, we achieved highest loading of 15 IOX per mAv by mole, which if enhanced can enable its use at higher concentrations. Additionally, our data show that a significant amount of uptaken mAv was retained within cartilage over 7 days, while CPC–IOX cleared out within 24 h (Figure 3). mAv–IOX was also uptaken by chondrocytes, while CPC–IOX was not. For high sensitivity, it is important that cellular uptake of the contrast agent is minimized^49^ and that it clears out of the joint following imaging. Both mAv and CPC at 2× higher working concentrations were safe to use as no changes in cell viability, metabolism, or effects on relative GAG loss and nitrite release from cartilage explants were observed compared to saline control. While mAv has been successfully used for delivery of small molecule drugs to cartilage in low doses,^20,23,24,50,51^ CT imaging requires many folds higher IOX doses compared to drugs. This may require mAv’s use at concentrations higher than 100 μM that have been shown to be safe.^27^ This makes use of mAv for CT imaging a less likely possibility unless its IOX loading efficiency is significantly improved.

## Conclusion

CPC–IOX offers itself as a safe and an effective cationic contrast agent that can enable (1) CT imaging of cartilage while distinguishing it from the subchondral bone and other soft tissues, (2) early OA diagnosis as it is sensitive in detecting early changes in GAG spatial distribution, and (3) also quantitative diagnosis of OA severity and its monitoring. For clinical feasibility, it is important that imaging can be done within a couple of hours following intra-articular administration of the contrast agent. Due to high partitioning at the cartilage interface, CPC–IOX can penetrate rapidly into cartilage in high concentrations even before equilibration is achieved. Thus, it has the potential to be used for non-equilibrium CT imaging, *i.e.*, within a couple of hours following its IA administration. Future work should focus on transient state CT imaging, especially at earlier time points both *in vitro* and *in vivo* using healthy and arthritic joints. Additionally, the *in vivo* environment is more complex with the presence of anionic synovial fluid and dynamic compression induced convective flow. While CPC is designed for preferential targeting of cartilage by minimizing its competitive binding with synovial fluid, it is imperative to test its use for non-invasive longitudinal cartilage imaging in animal models.

## Methods

### Materials

Ioxaglate (IOX), *N*,*N*′-disuccinimidyl carbonate (DSC), dimethylaminopyridine (DMAP), 3 kDa molecular weight cutoff (MWCO) Amicon centrifuge filter, thionyl chloride, fluorescein isothiocyanate isomer I (FITC), 4′,6-diamidino-2-phenylindole dihydrochloride (DAPI), and fluorescein diacetate (FDA) were purchased from Sigma-Aldrich (St. Louis, MO, USA). Anhydrous *N*,*N*-dimethylformamide (DMF), anhydrous pyridine, sodium bicarbonate, *N*-hydroxysuccinimido (NHS)–biotin, 7.0 kDa MWCO SnakeSkin dialysis tubing, trimethylamine (TEA), trifluoroacetic acid (TFA), acetonitrile, Avidin, and Avidin-Texas-Red conjugated, cyanine 5 (cy5)–NHS ester, chondroitinase ABC protease, 4% paraformaldehyde, and trypsin-EDTA were obtained from Thermo Fisher Scientific (Waltham, MA, USA). Cationic peptide carrier (CPC; peptide sequence, (RRAAAA)_3_RR) was synthesized using Fmoc solid-phase peptide synthesis (MIT Biopolymers and Proteomics, MIT, Cambridge, MA, USA). 10 kDa 8-arm poly(ethylene glycol) (PEG) amine was purchased from Advanced Biochemicals (Lawrenceville, GA, USA). Proteinase-K was purchased from Roche Diagnostics (Risch-Rotkreuz, Switzerland). High glucose Dulbecco’s modification of Eagle’s medium (DMEM) was from Cellgro (Manassas, VA, USA). HEPES, non-essential amino acids (NEAA), and penicillin–streptomycin antibiotic–antimycotic (PSA) were purchased from Gibco (Carlsbad, CA, USA). Ascorbic acid and l-proline were from Fisher Bioreagents (Pittsburgh, PA, USA). Propidium iodide (PI) was obtained from Thermofisher Acros Organics (Geel, Belgium).

### Cationic Peptide Carrier Conjugated with Ioxaglate (CPC-IOX)

CPC+8 ([RRAAAA]_3_RR) was synthesized by Fmoc solid-phase peptide synthesis using preloaded Wang resins (Life-Tein, Somerset, NJ, USA). The peptide was then purified by reverse-phase C18-HPLC (yielding >95% purity), and its mass was then confirmed by electrospray ionization–time-of-flight (ESI-TOF) mass spectrometry. The hydroxyl group of IOX was converted to reactive succinimidyl carbonate by introducing DSC. For this purpose,1 equiv of IOX (10.0 mg) was dissolved in 100 μL of DMF. 20 equiv of DSC (40.3 mg) dissolved in 200 μL of DMF and 10 equiv of DMAP (9.6 mg) dissolved in 200 μL of DMF were added to IOX solution dropwise and kept on stirring overnight at room temperature (RT). The intermediate product, IOX–succinimidyl carbonate (IOX–DSC) was precipitated in diethyl ether and washed using acetone for three times and was confirmed using matrix-assisted laser-desorption ionization–time-of-flight mass spectrometry (MALDI-TOF NS, Bruker Microflex II, Bruker, Billerica, MA, USA). IOX–DSC was lyophilized and used for reacting with the primary amine group of CPC to form a carbamate bond in CPC–IOX (Figure 1C). A 1 equiv amount of CPC (2.7 mg) and 1.5 equiv of IOX–DSC (2.5 mg) were reacted in 1 mL of PBS for 2 h. After reaction, CPC–IOX was purified using a centrifuge filter (3 kDa MWCO) to remove unreacted IOX–DSC.

### Multi-arm Avidin Conjugated with Ioxaglate (mAv–IOX)

First, a highly reactive acid chloride intermediate of IOX was formed by reacting its carboxyl group with thionyl chloride (Figure 1D). A 1 equiv amouont of IOX (12.7 mg) was suspended in 150 equiv of thionyl chloride (110 μL), and 5 μL of DMF was added to the mixture as a catalyst. The reaction temperature was increased to 70 °C to help with IOX solubility. After 6 h reaction, IOX–Cl was precipitated in ice-cold water (4 mL) and washed with saturated sodium bicarbonate solution and brine solution,^33^ and lyophilized. IOX–Cl was then conjugated to 10 kDa 8-arm PEG-amine using amide bond, which improved its solubility as well as its loading content for later conjugation to Avidin. First, 8-arm PEG was biotinylated as described before.^20^ A 1 equiv amount of 10 kDa PEG was reacted with 5 equiv of NHS–biotin for 2 h at RT. Excess NHS–biotin was removed using dialysis (7 kDa MWCO) for 24 h against PBS.

Thereafter, 1 equiv of 8-arm PEG-biotin (5.0 mg) dissolved in 100 μL of DMF was added dropwise to 10 equiv of IOX–Cl (6.4 mg) dissolved in 150 μL of DMF to synthesize biotin–PEG–IOX. Following addition of 7.5 μL of TEA, this reaction was kept shaking at 4 °C for 30 min and then at 50 °C for 16 h. Finally, the reacted solution was added to 4 mL of ice-cold water to precipitate and remove unreacted IOX–Cl. Supernatant solution was collected and lyophilized. The amount of IOX loaded on 8-arm PEGs was quantified by HPLC (Agilent Technologies 1260 infinity II) equipped with a variable wavelength detector using an Advance Bio RP-mAb C4 4.6 × 150 mm column. A gradient of solvent A (0.1% TFA in water) and solvent B (0.1% TFA in acetonitrile) was used. The concentration of solvent B was increased linearly from 5 to 65% over 26 min. Column temperature of 45 °C and a flow rate of 1.0 mL/min were used.

Finally, as described before,^20^ 4 equiv of biotin–PEG–IOX was mixed with 1 equiv of Avidin in DI water for 30 min at room temperature to synthesize mAv–IOX. Its hydrodynamic diameter and ζ potential were measured using a Particle Analyzer (Litesizer 500, Anton Paar, Austria).

### *In Vivo* Biodistribution of Cationic Contrast Agent Carriers

Animal studies were performed as preapproved by the Northeastern University Institutional Animal Care and Use Committee (NU-IACUC). Our previous work has shown that there is no cross-talk between the intra-articularly (IA) injected and the contralateral control knees;^52^ therefore, in this study design, both knees of animals were IA injected with treatment conditions. 50 μL of 1 mM cy5-labeled CPC or 50 μM Texas-Red-labeled mAv were IA injected through the patellar tendon into the joint space of left knees of healthy 8 to 10 week old Wistar male rats (Charles River Laboratories, Wilmington, MA, USA). The right knees were injected with an equivalent dose of free cy5 or Texas-Red as control conditions (Figure 2A). Both knees of two other rats were injected with only saline as untreated control. After administration, the rat joints were flexed and extended several times to ensure that the injected solute was well distributed throughout the intra-joint space. The *in vivo* dosages of CPC and mAv were determined based on carrier concentrations in 1 mg of I/mL of CPC-IOX and 0.5 mg of I/mL of mAv–IOX that demonstrated sufficient CECT signal *in vitro*.

A total of *N* = 4 joints per treatment condition were used for a total of 20 joints from 10 rats. After 6 h, rats were sacrificed, and the following tissues were extracted from each knee joint of treated and control animals: articular cartilage from femoral condyle (FC), tibial plateau (TC), and patella (PC); menisci (M); anterior and posterior cruciate ligaments (ACL and PCL); patellar and quadriceps tendons (PT and QT). These tissues were imaged using confocal microscope (LSM 800, ZEISS) to estimate depth of penetration and spatial distribution of cationic carriers at 10× magnification. 633 nm excitation/647 nm emission and 555 nm excitation/618 nm emission wavelengths were used for cy5 and Texas-Red, respectively. Z-stack imaging was applied to the *X*–*Y* plane of cartilage and ligament tissues to determine the depth of penetration of each solute. The menisci samples were sliced in the *X*–*Z* plane and imaged directly to determine the depth of penetration of each solute. Tissues were also weighed and digested using 1 mg/mL proteinase K, and fluorescence intensities were measured using a plate reader. Their glycosaminoglycan (GAG) content was also measured using the dimethyl-methylene blue (DMMB) assay.^53^

### Intra-cartilage Equilibration Time for Cationic Contrast Agents

With diameters at 3 mm and 1 mm thick (3 mm × 1 mm), cartilage explants with intact superficial zone were harvested from the femoropatellar grooves of 2 to 3 week old bovine knees (Research 87, Boylston, MA, USA) as explained previously.^15,54^ Cartilage explants were immersed in 300 μL of 0.5 mg of I/mL of IOX, 16 mg of I/mL of IOX, 0.5 mg of I/mL of CPC–IOX, and 0.5 mg of I/mL of mAv–IOX (*N* = 18 explants/group) in a 96-well plate at 37 °C. The osmolarity of contrast agent solution was adjusted to 400 ± 20 mOsm/kg using Micro Osmometer (Model 3300, Advanced Instruments, MA) by adding 10× PBS. At every time point of 0, 2, 4, 8, 12, and 24 h, three cartilage explants were taken out for sequential transaxial CT imaging using a μCT35 machine (SCANCO Medical AG, Switzerland) at an isotropic voxel resolution of 10 μm, 70 kVp tube voltage, 113 A current, and 200 ms integration time. The CT data sets were converted to DICOM format and processed in imageJ. The cartilage image was segmented by applying a threshold of −300 Hounsfield unit (HU) to distinguish the tissue position from the background (any signal lower than −300 HU was set as null). The mean CECT attenuation was calculated by averaging the CT attenuation of the whole cartilage explant over the entire segmented volume.

### Intra-cartilage Depth of Penetration

To fluorescently label IOX, 1 equiv of IOX dissolved in anhydrous pyridine and 5 equiv of FITC dissolved in anhydrous DMF were mixed at RT for 24 h (scheme is shown in Figure S4A).^55^ The FITC-labeled IOX was purified by HPLC equipped with a fraction collector and a variable wavelength detector, using a Poroshell 120 EC-C18, 4.6 mm × 150 mm column. A gradient of solvent A (0.1% TFA in water) and solvent B (0.1% TFA in acetonitrile) was used. The concentration of solvent B was increased linearly from 5 to 65% over 15 min. Column temperature of 30 °C and a flow rate of 1.0 mL/min were used. FITC-labeled IOX was eluted at 4.93 min at 280 nm wavelength (Figure S4B). A custom transport chamber (Figure 3B) was used to investigate the one-dimensional transport of cationic contrast agents from superficial zone (SZ) to deep zone (DZ) of cartilage as described previously.^19,54^ 6 mm × 1 mm cartilage half-discs were glued in the center of the chamber. The chamber compartment facing the SZ side of the cartilage was filled with 80 μL of 15 μM FITC-labeled IOX, CPC–IOX, or mAv–IOX, while the other chamber side was filled with PBS. This transport setup was placed in a Petri dish containing DI water to prevent evaporation and kept shaking at 37 °C for 6 and 24 h. Cartilage explants were sliced for confocal imaging (LSM 800, Zeiss).

### Equilibrium Uptake of Cationic Contrast Agents in Healthy and Arthritic Cartilage

3 mm × 1 mm cartilage explants were treated with 0.25 and 0.1 U/mL of chondroitinase ABC for 16 h to create 50 and 80% GAG depleted cartilage explants to simulate mid-stage and late-stage arthritic conditions, respectively. Explants were weighed and then equilibrated in 300 μL of 7.5 μM labeled IOX, CPC, CPC–IOX, mAv, and mAv–IOX (*N* = 6/group) in a 96-well plate for 6 h at 37 °C. The initial and equilibrated concentrations of the baths were measured using a plate reader (Synergy H1, Biotek). The equilibrium uptake ratios were calculated by dividing the solute concentration inside the cartilage by the concentration of solutes in the equilibrated bath.^19,54^ Following uptake experiments, the equilibrated cartilage explants were then desorbed in PBS in a 96-well plate for 7 days at 37 °C to estimate intra-cartilage retention using a plate reader.

### Chondrocyte Uptake Study

Primary chondrocytes were isolated from fresh bovine cartilage harvested from 2 to 3 week old bovine knees following methods described in our work.^56^ Isolated primary chondrocytes were seeded in a 48-well plate at the concentration of 100000 cells/well using complete chondrocyte culture media containing high glucose DMEM, 10% FBS, 1% HEPES, 1% non-essential amino acids (NEAAs), 1% penicillin–streptomycin antibiotic–antimycotic (PSA), 0.4% l-proline, and 0.4% ascorbic acid for 16 h. 200 μL of complete chondrocyte culture media and 5 μΜ FITC-labeled IOX, CPC, mAv, CPC–IOX, and mAv–IOX were added to each well (*N* = 6/group), respectively, for 2.5 h cultivation at 37 °C in a 5% CO_2_ environment. After treatment, the primary chondrocytes (*N* = 3/group) were washed with PBS twice and shaken with 200 μL of 4% paraformaldehyde for 10–15 min at 4 °C. The fixed cells were washed with PBS again and stained with 300 μL of 300 nM DAPI solution for 10 min and imaged using a fluorescence microscope (ECLIPSE Ts2R, Nikon, Japan). Separately, 200 μL of 0.25% trypsin–EDTA was added to a 48-well plate to detach chondrocytes (*N* = 3/group) for flow cytometry analysis (CytoFLEX, Beckman Coulter, CA). Cell suspensions were centrifuged at 1900*g*, 4 °C for 8 min; supernatants were removed, and cell pellets were replenished and washed with PBS at least 3 times. FITC signal in treated primary chondrocytes was acquired using flow cytometry. At least 1 × 10^4^ cells were analyzed for each sample.

### CECT Imaging of Cartilage Explants

3 mm × 1 mm cartilage explants were equilibrated in 300 μL of saline, IOX (1, 16, and 40 mg of I/mL), 1 mg of I/mL of CPC–IOX, and 0.5 mg of I/mL of mAv–IOX in a 96-well plate at 37 °C for 24 h. The osmolarity of contrast agent solution was adjusted to 400 ± 20 mOsm/kg. The CT data set of each cartilage explant was obtained by μCT35 machine as above. The data sets were imported to ImageJ, and cartilage stacks were segmented using a threshold^57^ of −300 HU. Cartilage stacks were then resliced to acquire a vertical section of cartilage with a superficial to deep zone; Z-projection method of average intensity was applied. Finally, a 16-color set in Look up tables (LUTs) was applied to create a color map of cartilage CT image with attenuation ranging from 0 to 700 HU. Representative images are shown for each treatment condition.

### Correlation between CT Attenuation and Cartilage GAG Density

3 mm × 1 mm cartilage explants were treated with 0.1 U/mL chondroitinase-ABC for 8 h at 37 °C to create arthritic cartilage samples with 80% GAG content and 0.25 U/mL chondroitinase-ABC for 16 and 24 h at 37 °C to create cartilage samples with 30 and 10% GAG content, respectively, to stimulate different OA stages (early, mid, and late stages); GAG content was determined using the DMMB assay. The GAG spatial distribution in cartilage was determined *via* staining with 0.5% Safranin O, 0.02% Fast Green, and Weigert’s iron hematoxylin. Explants were then equilibrated with contrast agents (16 mg of I/mL of IOX, 1 mg of I/mL of CPC–IOX, and 0.5 mg of I/mL of mAv–IOX) for μCT imaging. Cartilage GAG content was plotted against its respective mean CECT attenuation to estimate correlations.

### CECT Imaging of Intact Rat Tibial Bone

To delineate cartilage from other soft tissues and bone, intact tibial bones collected from 8–10 week old male Wistar rats (250–300 g) were equilibrated with contrast agents (1, 16, or 40 mg of I/mL of IOX, 1 mg of I/mL of CPC–IOX, and 0.5 mg of I/mL of mAv–IOX) or saline for 24 h. A total of 4 joints were used for each condition for a total of 24 joints from 12 rats. The tibial bones were imaged using μCT40 (SCANCO Medical AG, Switzerland) at an isotropic voxel resolution of 10 μm, 70 kVp tube voltage, 113 A current, and 300 ms integration time. The CT data sets were converted to DICOM format and processed in ImageJ. Cartilage stacks obtained from μCT40 were resliced to acquire sections of vertical planes of tibial bone. Rainbow colors in LUTs were added to images, and the minimum and maximum window levels were set from −1000 to 2800 HU. A 3D view of tibial bone was analyzed using a 3D Slicer image computing platform. The minimum and maximum window level were set −300 and 3500. Rainbow color in LUTs was added to 3D images. The attenuation less than −300 was set as background and shown in black color. Representative images are shown for each treatment condition.

### Chondrocyte Viability and Cartilage Health

3 mm × 1 mm fresh bovine cartilage explants were cultured with IOX (16 and 40 mg of I/mL), CPC (1 mM, which is the equivalent concentration needed to deliver 1 mg of I/mL of IOX in CPC–IOX and a high 2 mM dose), and mAv (50 μM, which is the equivalent dose required to deliver 0.5 mg of I/mL of IOX in mAv–IOX and a high 100 μM dose). Following 2 days treatment at 37 °C, explants were moved to fresh media without any contrast agents and cultured for another 6 days with media change at every 2 days. After day 8, chondrocyte viability in cartilage explants was analyzed by live–dead staining using FDA (4 mg/mL) and PI (10 mg/mL) and quantified using ImageJ. % cumulative GAG released to the media over 8 days of culture was estimated using the DMMB assay.^15,58^ The nitrite release to media from cartilage explants on day 2 and day 8 following treatment with contrast agents was measured using the Griess assay.^15^ At the end of the culture, cartilage explants were incubated with the media containing 10 μg/mL of resazurin sodium salt for 3 h at 37 °C and 5% CO_2_ (AlamarBlue assay). After incubation, the fluorescence of media was measured at 530 nm excitation and 590 nm emission wavelengths.

### Statistical Analysis

For all bovine cartilage explant studies, the general linear mixed effects model was used with animals as a random variable, followed by Tukey’s honesty significant different (Tukey’s HSD) test for comparisons between multiple treatment conditions. There was no effect on animal found and hence the data across animals were pooled. In general, *N* = 4–6 bovine cartilage explants per treatment condition from 5 to 6 animals were used for data presented in Figures 3, 4, and 6. The *N* = 4 rat joints per treatment condition was used for data shown in Figures 2 and 5. Data are presented as mean values ± standard deviation. *p* values less than 0.05 were considered statistically significant.

## Supporting Information Available

(Figure S1) MALDI-TOF mass spectrometry and HPLC data of IOX-DSC; (Figure S2) ^1^H NMR spectra, synthesis scheme, MALDI-TOF mass spectra, and mAv characterization; (Figure S3) intra-cartilage retention of CPC and mAv; (Figure S4) synthesis scheme and HPLC confirmation for IOX-FITC; (Figure S5) total GAG content in cartilage explants after culture (PDF)
