# Supplementary material for: Cationic Carrier Mediated Delivery of Anionic Contrast Agents in Low Doses Enable Enhanced Computed Tomography Imaging of Cartilage for Early Osteoarthritis Diagnosis
Source: ACS Nano. 2023 Mar 29;17(7):6649–63. doi: 10.1021/acsnano.2c12376 (PMC10629240; doi:10.1021/acsnano.2c12376)
Supplement: Supplementary file 1 — nn2c12376_si_001.pdf [file nn2c12376_si_001.pdf]

Supporting Information

Cationic Carrier Mediated Delivery of Anionic Contrast Agents in Low Doses Enable Enhanced Computed Tomography Imaging of Cartilage for Early Osteoarthritis Diagnosis

*Chenzhen Zhang[a], Armin Vedadghavami[a], Tengfei He[a], Julia F. Charles[b], Ambika G. Bajpayee[a,]**

[a]Department of Bioengineering, Northeastern University, 360 Huntington Ave, Boston, MA 02115, United States

[b]Department of Orthopaedic Surgery, Brigham and Women's Hospital, 60 Fenwood Rd, Boston, MA 02115, United States

* Email: a.bajpayee@northeastern.edu

Corresponding Author:

Ambika G. Bajpayee

Phone: (617)-373-7018; Email: a.bajpayee@northeastern.edu

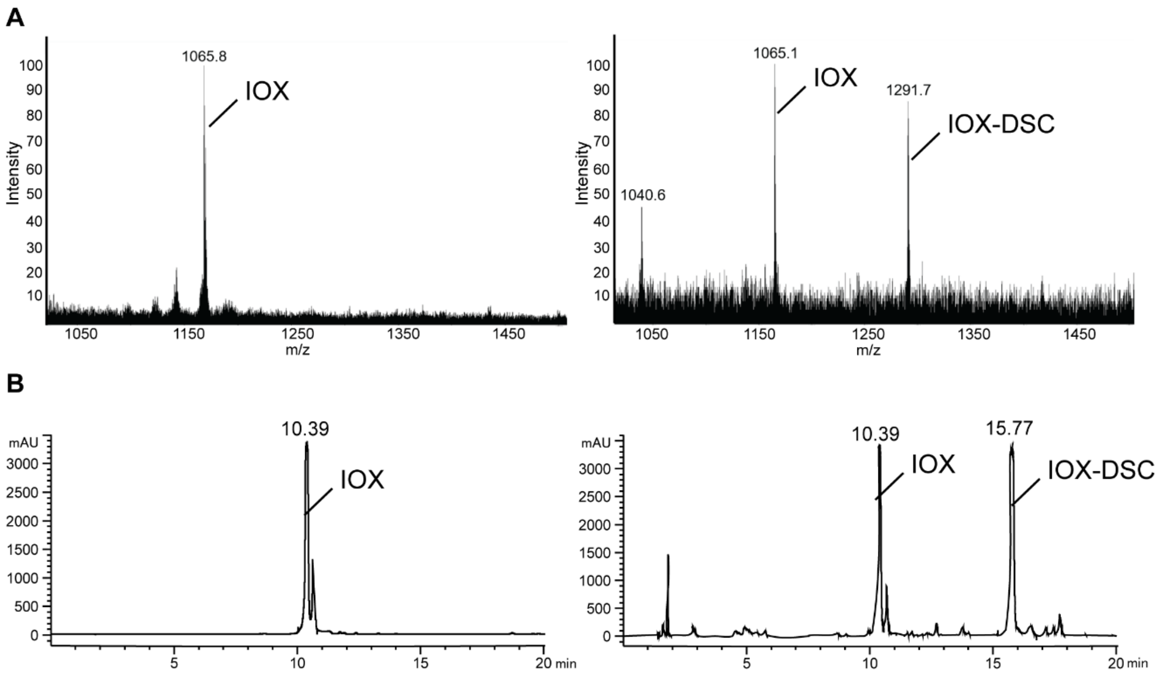

Figure S1A. MALDI-TOF mass data confirmed the increased mass of IOX from 1065.8 Da to 1291.7 Da after reacting with DSC. **B.** HPLC confirmed the new peak of IOX-DSC at 15.773 min shifting from 10.387 min (IOX peak).

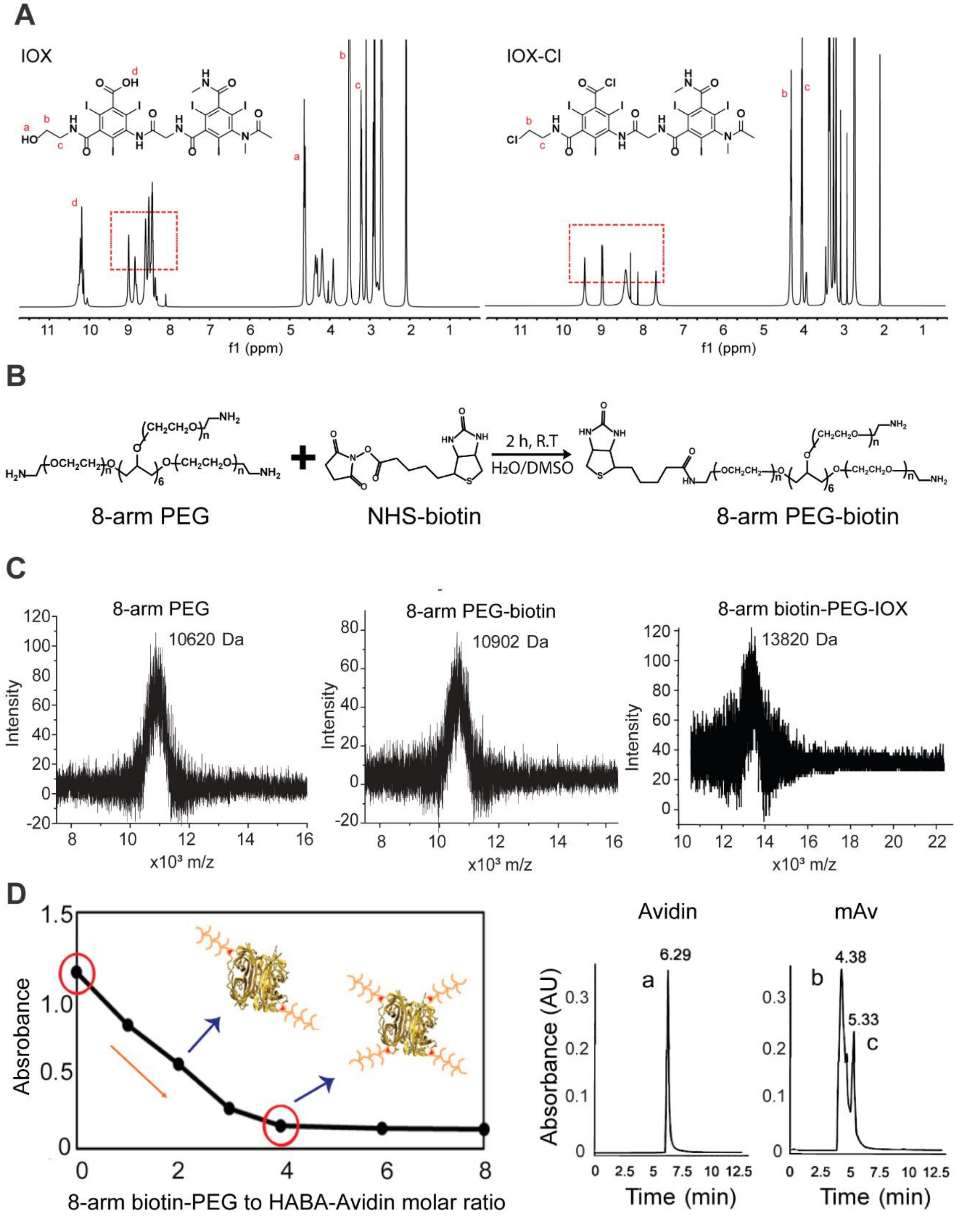

Figure S2A. ^{1}H-NMR confirmed the structure of IOX and IOX-Cl. **B.** Scheme showing synthesis of 8-arm PEG-biotin. MALDI-TOF data for **C.** 8-arm PEG and 8-arm PEG-biotin shows increased mass of 282 Da implying about 1.15 biotin conjugated to per mole of 8-arm PEG. 8-arm PEG-IOX was at 13820 Da confirming an average of 2.33 moles of IOX conjugated to one mole of 8-arm biotin-PEG. **D.** Titration curve of 8-arm PEG-biotin with HABA-Avidin mixture (left). Absorbance value dropped with increasing 8-arm PEG-

biotin : HABA-Avidin molar ratio, and a plateau was achieved at the 4:1 molar ratio, confirming that all four biotin binding sites of Avidin were occupied by 8-arm PEG-biotin to form mAv. UPLC analysis (right) also confirmed the structure of mAv formulation containing a majority of mAv with 4 PEGs (peak ‘b’ at 4.38 min) and a secondary population of mAv with 2 PEGs (peak ‘c’ at 5.33 min), where native Avidin was eluted later at 6.29 min (peak ‘a’). Reprinted with permission from ref 1. Copyright 2020 Elsevier.

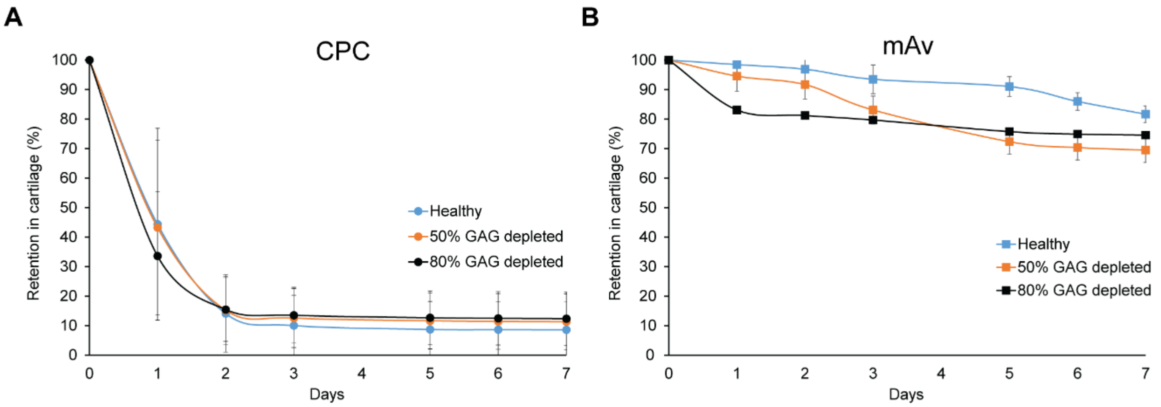

Figure S3. % Retention of **A.** CPC and **B.** mAv in healthy, 50% GAG depleted and 80% GAG depleted cartilage samples when desorbed in PBS over 7 days.

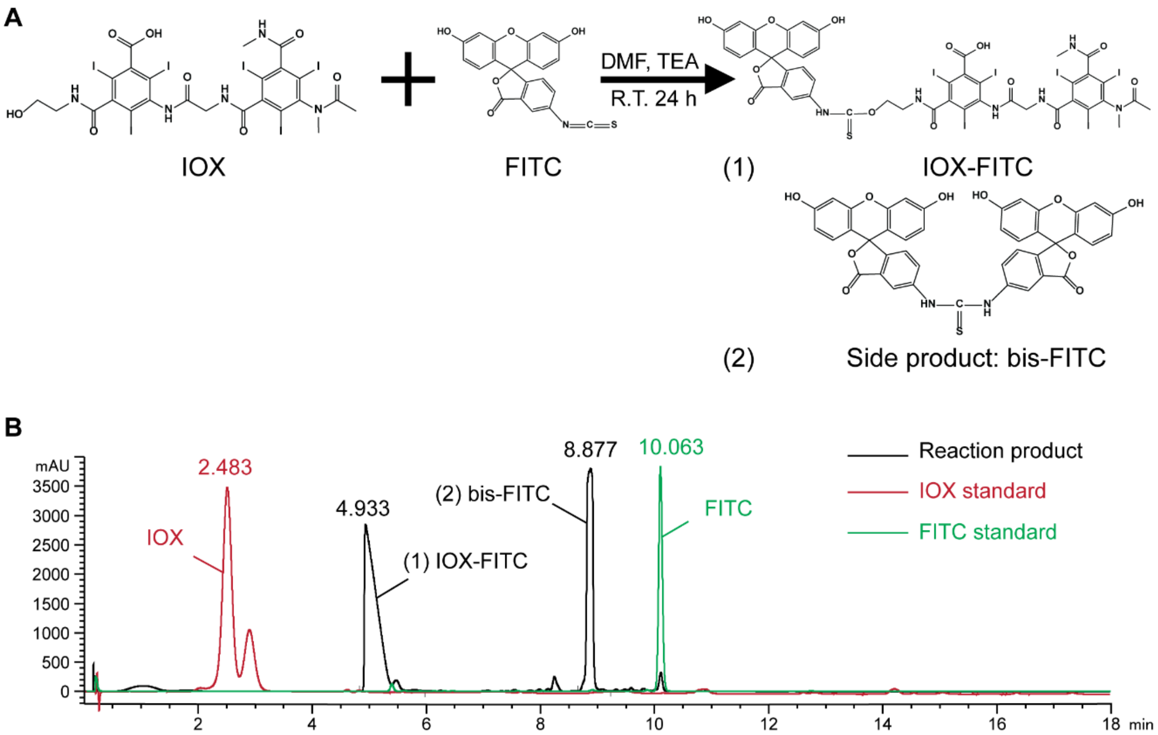

Figure S4A. Schemes showing (1) FITC labeling of IOX and its side product (2) bis-FITC. **B.** HPLC revealed that IOX eluted peak at 2.483 min, FITC peak at 10.063 min, FITC labeled IOX at 4.933 and bis-FITC at 8.877 min.

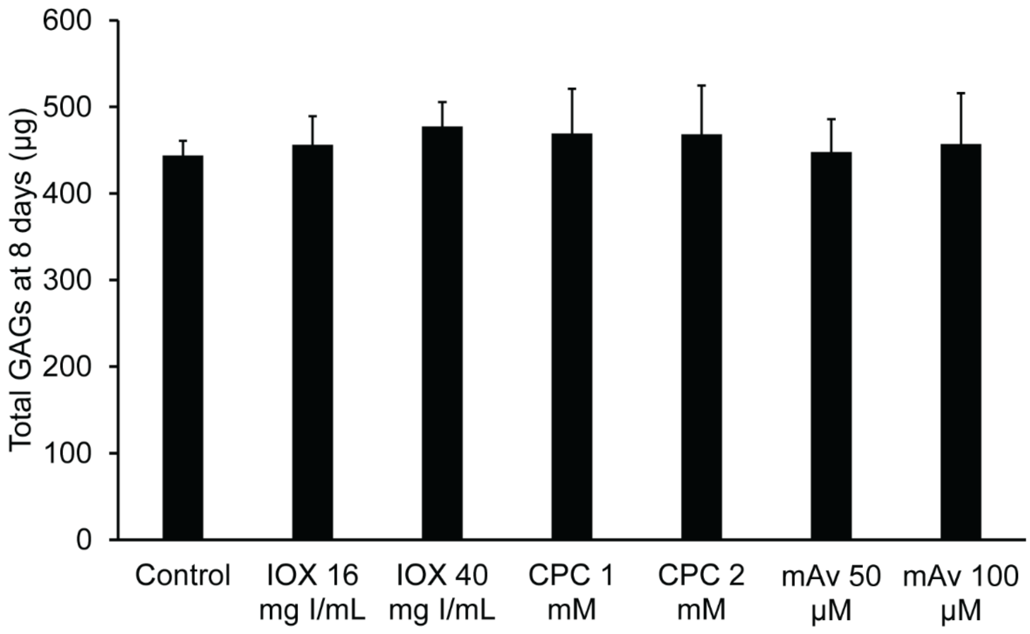

Figure S5. Total GAG content in cartilage explants at the end of culture. This is estimated by summing the total GAGs released to media and that remaining within the cartilage tissue at the end of 8-day culture.

References

1. He, T.; Zhang, C.; Vedadghavami, A.; Mehta, S.; Clark, H. A.; Porter, R. M.; Bajpayee, A. G., Multi-arm Avidin nano-construct for intra-cartilage delivery of small molecule drugs. *J Control Release* **2020,** *318*, 109-123.
